# Flexible thermoelectric generators from spray-printed PEDOT:PSS/Bi_0.5_Sb_1.5_Te_3_ composites†

**DOI:** 10.1039/d4ra08450k

**Published:** 2025-02-27

**Authors:** Saeed Masoumi, Ruifeng Xiong, Eoin Caffrey, Riley Gatensby, Cansu Ilhan, Jonathan N. Coleman, Amir Pakdel

**Affiliations:** a Department of Mechanical, Manufacturing, and Biomedical Engineering, Trinity College Dublin, The University of Dublin Dublin D02PN40 Ireland pakdela@tcd.ie masoumis@tcd.ie Xiongru@tcd.ie; b CRANN & AMBER Research Centres, Trinity College Dublin, The University of Dublin Dublin D02PN40 Ireland eocaffre@tcd.ie gatensbr@tcd.ie ILHANC@tcd.ie colemaj@tcd.ie; c School of Physics, Trinity College Dublin, The University of Dublin Dublin D02PN40 Ireland; d School of Chemistry, Trinity College Dublin, The University of Dublin Dublin D02PN40 Ireland

## Abstract

Energy harvesting technologies play a pivotal role in powering the next generation of wearable and portable devices, where thin-film thermoelectric generators (TEGs) offer a compact and flexible solution. In this study, flexible thin films of poly(3,4-ethylenedioxythiophene)polystyrene sulfonate (PEDOT:PSS)/Bi_0.5_Sb_1.5_Te_3_ composite on flexible polymeric substrates were initially developed using a spray printing technique. The effect of substrate temperature during printing was assessed on the microstructural and thermoelectric properties, yielding a maximum power factor at a substrate temperature of 110 °C. Additionally, the printed films demonstrated excellent flexibility and mechanical/electrical stability during 1000 cycles of bending, confirming their suitability for wearable electronic applications. Subsequently, a flexible thin-film TEG containing 40 thermoelectric legs was fabricated by spray printing of the composite ink for the first time. Finally, the electrical performance of the flexible thin-film TEG was thoroughly assessed under various temperature gradients, exhibiting maximum open circuit voltage of 52 mV at a temperature difference of 50 °C. This study establishes a foundation for the facile fabrication of flexible TEGs using organic/inorganic composite inks. Further enhancement of the thermoelectric performance can be envisaged through post-processing chemical treatments to optimize charge carrier concentration in the printed TEGs.

## Abstract 2

Energy harvesting technologies play a pivotal role in powering the next generation of wearable and portable devices, where thin-film thermoelectric generators (TEGs) offer a compact and flexible solution.

## Graphical abstract

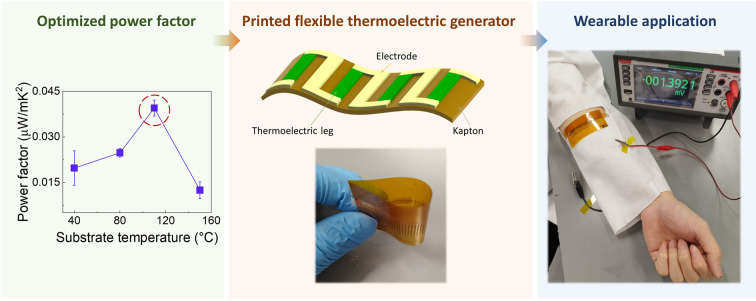

## 1 Introduction

The Internet of Things (IoT) enables communication among sensor-embedded objects *via* the internet, and for continuous operation, it requires a reliable power supply. Integrating energy harvesters like photovoltaic devices, piezoelectric generators, and thermoelectric generators (TEGs) allows IoT devices to be self-powered. However, each energy harvesting technology has certain operational constraints; for instance, photovoltaic devices require sunlight and piezoelectric generators need constant mechanical forces.^1^ TEGs, however, can directly convert thermal energy into electrical energy in the presence of a temperature gradient (Δ*T*). The conversion efficiency of a thermoelectric (TE) material is closely related to a dimensionless figure of merit 
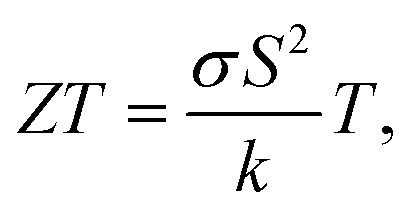
 where *σ* is the electrical conductivity, *S* is the Seebeck coefficient, *k* is the thermal conductivity, and *T* is the absolute temperature.^2^ The TE power factor, PF = *σS*^2^, is also regularly used to evaluate the electrical performance of TE materials. The output power of a TEG strongly depends on Δ*T*, the TE properties of the constituent materials, and the fabrication method.^3,4^

In recent years, flexible TE materials and devices have attracted attention due to the widespread use of wearable electronics in IoT devices.^5^ An example of flexible TE materials is semiconducting polymers, which have demonstrated great mechanical flexibility, intrinsically low thermal conductivity, and moderate electrical conductivity and Seebeck coefficient.^5–7^ Among them, poly(3,4-ethylenedioxythiophene):poly(styrenesulfonate) (PEDOT:PSS) has been popular for TE application at around room temperature.^8^ To improve the TE performance of PEDOT:PSS, various approaches have been used including chemical doping, modification of molecular structure, and hybridization with inorganic semiconductors.^9^ Such organic/inorganic composites are particularly interesting because they combine the flexibility and low thermal conductivity of organic semiconductors with the superior power factor of inorganic materials such as Bi_2_Te_3_-based compounds and CuI.^10–13^ Significant advancements in the design and functionality of such flexible TE materials have been achieved, which also paved the path for developing various types of flexible TEGs using conventional fabrication methods such as drop casting,^14^ wet-spinning,^15,16^ and vacuum filtration.^17–19^ However, these techniques possess certain limitations such as low-resolution patterning for producing miniature-sized TEGs, and complexity for large-scale production of high-performance flexible TEGs.^20^ Modern production technology such as printing enable cost-effective, high-resolution, and large-scale production of flexible TE devices.

Various types of printing methods, including dispenser printing,^21,22^ spray printing,^23,24^ screen printing,^25–29^ aerosol jet printing,^30,31^ and inkjet printing,^32^ have been used for developing flexible TEGs made of inorganic, organic, and hybrid TE materials. There have also been advancements in TE ink formulations and post-treatment processes to improve the TE conversion efficiency.^33,34^ Printed inorganic TEGs achieved higher output power, but their flexibility was often constrained.^21,28^ Organic TEGs based on PEDOT:PSS exceled in mechanical flexibility and adaptability for wearable electronics, but generally have lower output power.^22,24,35^ Therefore, composite-based TEGs were developed aiming to combine the flexibility of polymers with the better TE performance of inorganic materials such as Bi_2_Te_3_,^29^ Sb_2_Te_3_,^30^ and Cu_2_S.^27^ Additionally, when these materials are used for fabrication of flexible films and devices, they can maintain their TE properties under mechanical loading and deformation. Reports show less than 10% changes in *σ* and *S* of PEDOT:PSS films after hundreds of bending cycles.^14,27,36–39^ A summary of printable and flexible p-type organic, inorganic, and organic/inorganic composite films, TE materials, and TEGs – particularly those based on PEDOT:PSS and bismuth telluride compounds – is presented in ESI Table S1.†^20,33,40–42^ Most of those studies have used dispenser printing, inkjet printing, and aerosol jet printing which allow for direct additive manufacturing of TE elements with different patterns. However, they are particularly limited by challenges in formulating stable TE inks. Some studies have utilized screen printing to develop TE films and TEGs with larger thicknesses, benefiting from higher temperature differences in the vertical direction. However, most studies have not investigated the mechanical performance and TE properties of the developed flexible films after application of cyclic loading.

To the best of the authors' knowledge, there is no study on the development of flexible hybrid TEGs made of PEDOT:PSS and Bi_2_Te_3_ particles using spray printing technique. Additionally, the effect of printing parameters (other than ink formulation) has not been systematically studied to optimize the conversion efficiency of the flexible TEGs. In this article, flexible PEDOT:PSS/Bi_0.5_Sb_1.5_Te_3_ composite thin films are fabricated on flexible polymeric substrates using a spray printing technique. The effect of substrate temperature on microstructure and TE properties of the films is investigated to optimize the deposition temperature. Additionally, durability of the TE films is studied by measuring their electrical properties after 1000 cycles of bending and stretching. Then a flexible TEG made of 40 TE legs is fabricated through spray printing of a TE ink containing PEDOT:PSS/Bi_0.5_Sb_1.5_Te_3_ pre-treated with dimethyl sulfoxide (DMSO) and sodium hydroxide (NaOH) for tailoring its structural morphology and doping level. The electrical properties of the flexible TEG are then characterized, and it is attached to the human arm to generate voltage from the body heat.

## 2 Materials and methods

### 2.1 Materials

A highly conductive PEDOT:PSS suspension (1.0 wt% in H_2_O, high-conductivity grade), DMSO (99.9%), NaOH (≥98%), hydrochloric acid (HCl; ≥37%), ethanol (99.99%), and silver paste (1.59 μΩ cm, 20 °C) were purchased from Sigma-Aldrich. A Bi_0.5_Sb_1.5_Te_3_ (BST) ingot was purchased from Thermonamic Electronics (Jiangxi) Corp., Ltd. Glass microscope slide was purchased from VWR International LLC. Nichrome wire was purchased from RS PRO. The Kapton sheet was purchased from DuPont de Nemours, Inc.

### 2.2 Spray printing of PEDOT:PSS/BST thin films

**Fig. 1:**
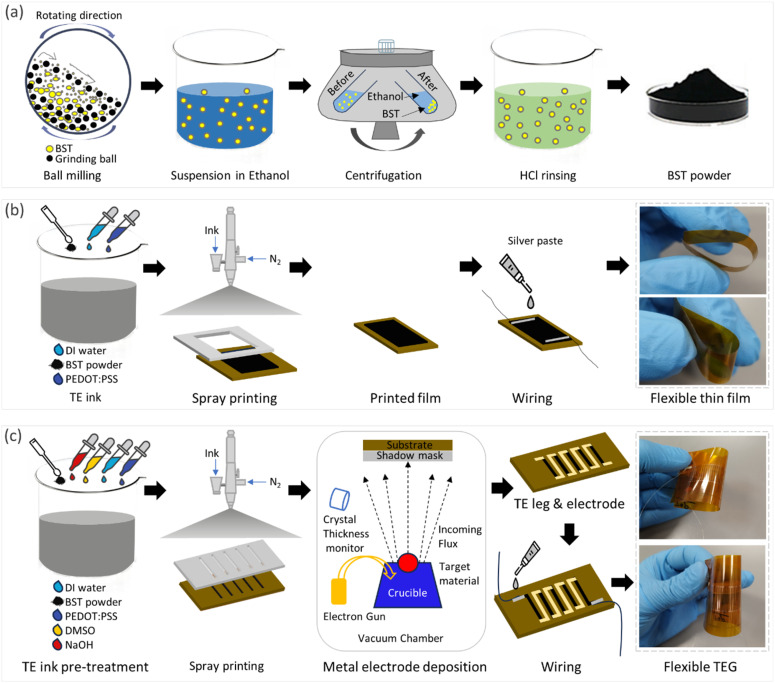
(a) Ball milling and centrifugation processes to prepare BST powder. (b) The spray printing fabrication of flexible TE thin films from an aqueous PEDOT:PSS/BST ink. (c) The printing and deposition processes used to produce flexible TEGs.

BST powder was produced through a three-step process. Initially, the BST ingot was crushed and ground, followed by ball milling (Retsch Planetary Ball Mill, PM 100), and finally centrifugation (Eppendorf, Centrifuge 5804). During ball milling, 1200 zirconium oxide balls of 5 mm diameter were used at a speed of 600 rpm for 20 hours, with 15 minute intervals to prevent excessive powder heating. The milling jar had a capacity of 250 mL and was fed with 190 g of BST powder. After milling, a suspension of the BST powder in ethanol was prepared with a concentration of 50 mg mL^−1^, followed by 4 hour sonication in an ultrasonic bath (Elma Schmidbauer GmbH, TI-H-10). Then, BST powder with an average particle size of ∼1.4 μm was obtained through centrifugation at 500 rpm and subsequently rinsed with an HCl solution (5% HCl and 95% DI water) to remove any potentially formed oxide layers. The fabrication process of the BST powder is illustrated in Fig. 1a. Scanning electron microscopy (SEM) images, and particle size distribution graphs of the BST powders after ball milling and centrifugation are presented in ESI Fig. S1.† All the aforementioned process parameters were selected based on the authors' previous systematic study of the TE properties of PEDOT:PSS thin films hybridized with BST particles. The influence of BST particle size and concentration, and the effect of various chemical post-treatment strategies on the TE performance of PEDOT:PSS/BST composites thoroughly discussed in that research.^10^

In the next step, a PEDOT:PSS suspension with 40 wt% BST powder was prepared, as this concentration was identified optimal in authors' previous work.^10^ To create a suitable ink for spray printing, the suspension was diluted with 95 vol% DI water, followed by 1 h of bath sonication. Then, flexible Kapton sheets were used as substrates for spray printing of the composite. The TE inks were used for spray printing *via* a Harder and Steenbeck Infinity airbrush connected to a computer-controlled Janome JR2300N mobile gantry.^43^ A nitrogen back pressure of 310 MPa, nozzle diameter of 0.4 mm and stand-off distance of 100 mm between the nozzle and substrate were used for the spray printing process. A deposition rate of 1 nm min^−1^ was used to prepare films with a thickness of 0.1 mm and a lateral size of 25 mm × 15 mm. A total volume of 2 mL of ink was utilized for printing each sample. The spray printing process took place at various substrate temperatures, including 40 °C, 80 °C, 110 °C, and 150 °C. For electrical characterization of the resulting thin films, Ni–Cr wires were connected to them using a silver paste. For comparison, the same spray printing procedure was also tested on glass substrates. A schematic of the fabrication process and images of the printed flexible films are depicted in Fig. 1b. The full printing procedure and examples of printed films on rigid and flexible substrates are illustrated in ESI Fig. S2.†

### 2.3 Spray printing of flexible TEGs

Flexible TEGs were fabricated through spray printing on a Kapton substrates of 95 mm × 45 mm dimensions. They consisted of 40 legs, each with a width of 200 μm and a length of 20 mm, and electrically connected in series by metal electrodes. The width and length of the electrodes were similar to the TE legs, and a space of 200 μm was maintained between them. The 200 μm spacing between TE legs and metal electrodes was chosen because the minimum feature size achievable during the fabrication of shadow masks by CNC machines was 200 μm. While larger width of TE legs and spacing between them could simplify the fabrication process, 200 μm was selected to maximize the number of TE legs in a given area to enable a higher open-circuit voltage in the TEG. Two types of shadow masks, one for printing TE legs and another for metalization, were utilized during TEG fabrication, as depicted in ESI Fig. S3.† This process involved four main stages: pre-treatment of the ink, printing TE legs, deposition of electrodes, and wiring, as schematically shown in Fig. 1c. In the first step, the aqueous suspension of PEDOT:PSS and BST underwent pre-treatment with 10 vol% DMSO and 0.1 M NaOH. For the second step, the printing process was conducted with the substrate temperature set to 110 °C, and the shadow mask was placed on top of the substrate. The printing parameters remained similar to those outlined in Section 2.2. In the third stage, metal electrodes were deposited for connecting the TE legs using an electron beam evaporation system while the shadow mask was placed on top of the substrate. These electrodes consisted of 20 nm of gold on 2 nm of titanium. Finally, Ni–Cr wires were connected to the TEG by a silver paste. An example of fabricated flexible TEGs is shown in Fig. 1c.

### 2.4 Materials and device characterization

Surface morphology of the fabricated thin films were analyzed using a field-emission scanning electron microscope (FE-SEM; Carl Zeiss Ultra) and an atomic force microscope (AFM; Asylum MFP-3D). The AFM was operated in non-contact mode, using a silicon microcantilever probe with an ambient force constant of 42 N m^−1^. The thickness of the films was also measured using the AFM. For chemical characterization the composite, X-ray diffraction (XRD) was performed using a PanAlytical X'Pert Pro diffractometer with Cu Kα radiation (1.5406 Å).

**Fig. 2:**
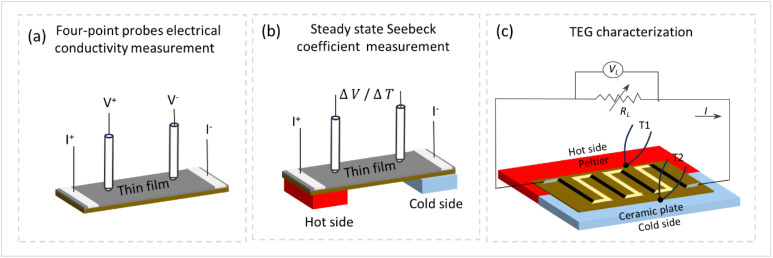
Schematic illustration of (a) electrical resistance measurement, (b) Seebeck coefficient measurement, and (c) TEG characterization setup.

An automated four-point probe system was used for measuring the Seebeck coefficient and electrical conductivity of the samples.^44–47^ Temperature gradients were created by two independently controlled microheaters on each side of a sample, and were measured by two K-type thermocouples placed on top of the sample at the cold and hot ends. The thermocouples had a Seebeck coefficient of 41 μV K^−1^ above room temperature. The Seebeck coefficients of the thermocouple legs were compensated after each measurement to obtain the absolute Seebeck coefficient value for the thin films. Schematics of electrical resistance and Seebeck coefficient measurement principles are depicted in Fig. 2a and b. A photograph of the apparatus is presented in ESI Fig. S4.† To evaluate the mechanical and electrical stability of the flexible thin films, changes in relative conductivity and Seebeck coefficient were measured at various bending radii and cycles, while maintaining a fixed temperature gradient.

A purpose-built experimental setup was devised to evaluate the electrical efficiency of the TEGs, as schematically shown in Fig. 2c. It comprised two commercial Peltier modules with dimensions of 4 cm × 4 cm, which were used to heat one side of the TEG, while the other side was kept at room temperature. Two thermocouples were used to measure the temperature on the hot and cold sides. The open circuit voltage of the TEG was measured at various temperature gradients, using a digital multimeter (Keithley, DMM6500). The TEG was connected to a load shunt variable resistor (General Radio Co/Decade Resistor) for impedance matching. The load voltage from the TEG was recorded at different temperature gradients. The current and output power were calculated using the measured load voltage and shunt resistance. The experimental setup is presented in ESI Fig. S5.†

## 3 Results and discussion

### 3.1 Morphological, optical, and thermoelectric properties of printed thin films

Composite thin films were printed at different substrate temperatures of 40 °C, 80 °C, 110 °C, and 150 °C. The test samples of thin films on glass substrates are presented in ESI Fig. S2c–f.† It was observed that films deposited at lower temperatures were not uniform, but those printed at 110 °C and 150 °C were homogeneous, as shown in ESI Fig. S2e and f.† This can be attributed to drying characteristics of the TE ink on the substrate. The low substrate temperatures were insufficient to fully evaporate the water solvent in the TE ink after one layer of spray printing. As a result, some of the sprayed TE ink remained in a liquid phase, and spraying the next layer disturbed the uniformity of the previous layer.

**Fig. 3:**
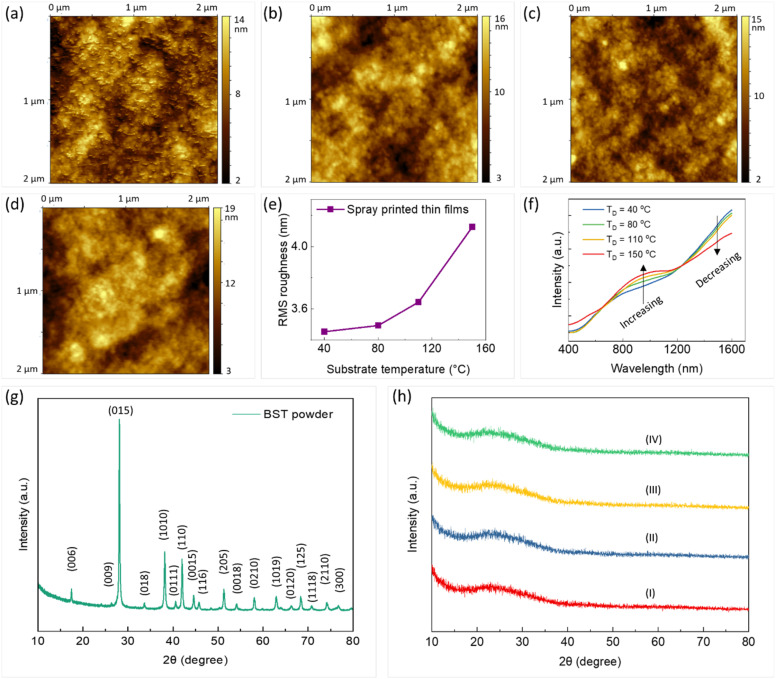
AFM surface topography images of PEDOT:PSS/BST thin films printed at different temperatures on glass substrates: (a) 40 °C, (b) 80 °C, (c) 110 °C, and (d) 150 °C. (e) RMS roughness of the composite thin films *vs.* substrate temperature, (all the surface scan areas for topography images were: 2 × 2 μm^2^). (f) Optical absorption spectra of composite thin films printed at different substrate temperatures. (g) XRD pattern of BST powder, (h) XRD spectra of printed thin films on glass substrates: PEDOT:PSS at 40 °C (I), PEDOT:PSS/40 wt% BST at 40 °C (II), 110 °C (III), and 150 °C (IV).

The AFM surface topology of the printed composite thin films is shown in Fig. 3a–d. The PEDOT:PSS/BST thin films exhibited a relatively smooth and uniform surface with no obvious phase separation in the structure. As shown in Fig. 3e, the Root Mean Square (RMS) roughness of the films printed at 40 °C, 80 °C, 110 °C, and 150 °C were 3.45 nm, 3.49 nm, 3.64 nm, and 4.13 nm, respectively. Therefore, raising the substrate temperature did not change the RMS roughness considerably (*i.e.*, only a slight increase), similar to previous reports in the literature.^48^ AFM step-height profiles were used to calculate the thickness of all the composite thin films, as shown in ESI Fig. S6.† The average thickness of the composite films printed above 80 °C were in the range of 108–145 nm, while the average thickness of the sample printed at 40 °C was considerably higher (227 nm) due to its non-homogeneity. The acquired thicknesses were used to calculate the electrical conductivity of the films. Fig. S7 in ESI† displays typical SEM micrographs of PEDOT:PSS thin films containing 0 and 40 wt% BST particles. It is evident that particle distribution is homogeneous in the composite film.

**Fig. 4:**
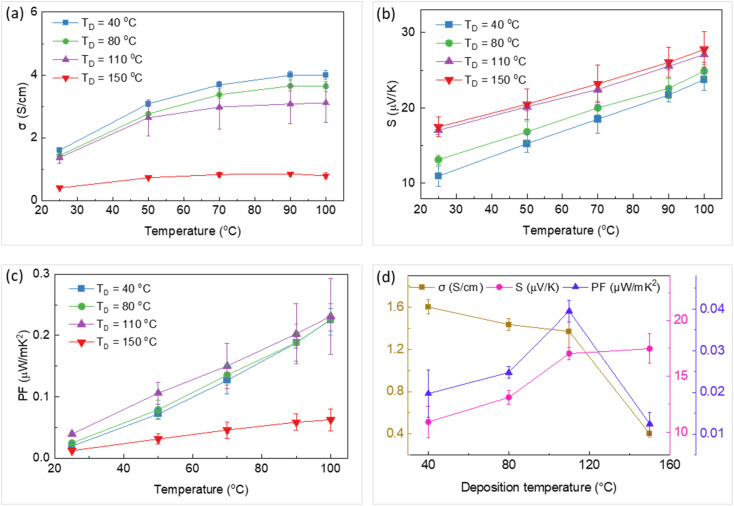
Temperature dependency of (a) electrical conductivity, (b) Seebeck coefficient, and (c) power factor of the thin films printed at different substrate temperatures (*T*_D_). (d) The variation of electrical conductivity, Seebeck coefficient, and power factor with deposition temperature. The error bars were calculated by two times repetition of measurements on each sample.

The optical absorption spectra of PEDOT:PSS thin films can be employed to characterize chemical changes induced by chemical dedoping.^49^ The three states of PEDOT chains, namely bipolaron, polaron, and neutral states, exhibit absorption at different wavelengths. Neutral polymer chains show absorption around 600 nm, chains in the polaron state exhibit absorption around 900 nm, and chains in the bipolaron state display broad absorption in the near-infrared region.^50^ The optical absorption spectra of the printed PEDOT:PSS/BST films are shown in Fig. 3f. The increase in substrate temperature from 40 °C to 150 °C led to a decrease in the bipolaron absorption region and an increase in the polaron absorption region in the films. At a substrate temperature of 150 °C, a shoulder below 600 nm appeared, which represents the neutral state. These results demonstrate the change in the oxidation state of the films from bipolaron to polaron and neutral states, and indicate a change in charge carrier concentration (as will be discussed further in Fig. 4). This trend is consistent with the previously reported literature.^51^

To analyze the effect of substrate temperature on the overall structure of the composite thin films, XRD characterization was performed on the BST powder, pristine PEDOT:PSS and PEDOT:PSS/40 wt% BST films printed at 40 °C, 110 °C, and 150 °C. As shown in Fig. 3g and h, the diffraction peaks of the powder can be indexed as rhombohedral structured BST with a space group of *R*3̄*m* and the main diffraction peak at 2*θ* = 28.3° corresponding to the (015) lattice plane (JCPDS no. 49-1713). However, the polymeric printed thin films showed a broad peak at 2*θ* of ∼20–32°, attributed to interchain planar stacking of PEDOT planes.^52–54^ The weak intensity and broad diffraction peak are due to the small thickness of the films and also moderate crystallinity, consistent with the literature.^10,17,54^ The XRD patterns did not show a notable change after addition of BST particles or changing the printing temperature. Considering that 40 wt% BST corresponds to a much smaller vol% BST in the composite (the density of PEDOT:PSS is more than 7 times smaller than that of BST), the lack of sharp BST peaks in Fig. 3h could be due to the low vol% of these particles in the composite. Similar observations have been reported previously in case of such thin composite films (10 s of nm in thickness).^17,55,56^ However, studies on micrometer-thick and free-standing composites of PEDOT:PSS/BST have demonstrated distinguishable diffraction peaks due to larger mass of each sample.^17,23,55–57^

In the next stage, composite thin films were printed on flexible Kapton substrates. The electrical conductivity, Seebeck coefficient, and power factor of these films printed at 40 °C, 80 °C, 110 °C, and 150 °C are shown in Fig. 4a–c. These properties were recorded at different temperatures (between 25 °C and 100 °C) in all samples. Both the electrical conductivity and Seebeck coefficient increased with the temperature in all cases. This simultaneous increase can be described by Mott's variable range hopping (VRH) transport mechanism, in which 
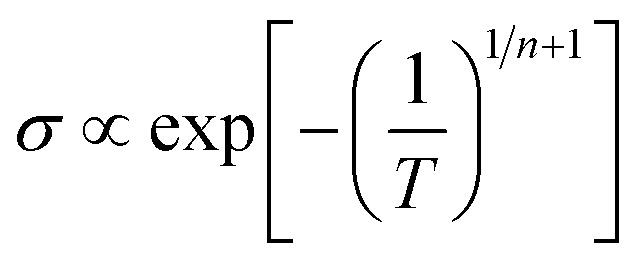
 and *S* ∝ *T*^*n*−1/*n*+1^. Experimental and theoretical studies suggest that the *n* value in PEDOT:PSS films is 3;^48,58,59^ thus, both *σ* and *S* could increase with temperature. This behaviour highlights the role of particle-to-particle hopping in the composite. These trends of variation are consistent with data reported in the literature.^51,60,61^ The effect of substrate temperature during printing on the TE properties of these samples at room temperature is illustrated in Fig. 4d. The electrical conductivity of the thin films decreased with deposition temperature, while the Seebeck coefficient increased. This result can be attributed to a change in the oxidation state of the PEDOT from bipolaron to polaron and neutral states as the substrate temperature increased from 40 °C to 150 °C. The optical absorption of the four thin films shown in Fig. 3f supports the change in PEDOT oxidation states. Power factors of 0.04 μW mK^−2^ and 0.23 μW mK^−2^ at room temperature (25 °C) and 100 °C were achieved for the thin film printed at 110 °C. Additionally, this thin film showed a uniform morphology with no traces of splashing, coffee-stain effect, and wetting/drying issues that can arise during a printing process. Consequently, the substrate temperature of 110 °C was deemed suitable for printing TEGs in the next stage.

### 3.2 Mechanical stability and flexibility of printed thin films

**Fig. 5:**
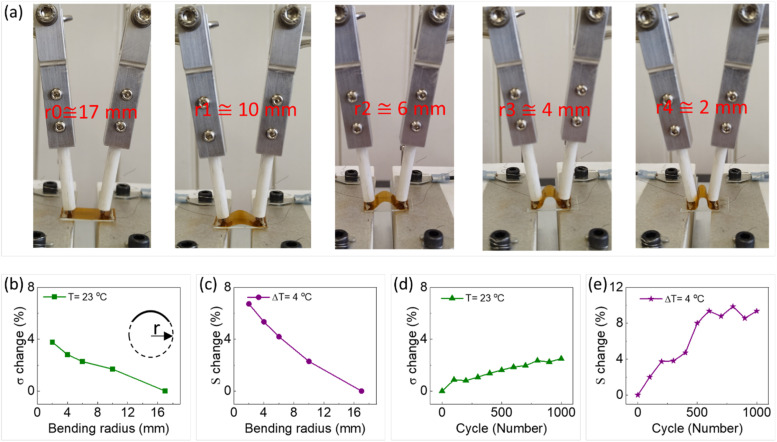
(a) Images of a composite thin film printed on a Kapton substrate at 110 °C under different bending radii to test the mechanical flexibility and stability. The relative changes of (b) electrical conductivity and (c) Seebeck coefficient in response to different bending radii after 500 bending cycles. The relative changes of (d) electrical conductivity and (e) Seebeck coefficient in response to the bending cycles for a bending radius of 4 mm. The inset in (b) schematically shows how bending radius is defined.

It is important for flexible TE materials and devices to maintain their good TE performance under mechanical loading and deformation. To this end, a PEDOT:PSS/BST thin film was spray printed on a Kapton substrate at 110 °C and then bent alternately. The relative electrical conductivity and Seebeck coefficient changes were measured at different bending radii and cycles. The tests were performed at an average temperature of 25 °C, and the temperature gradient along the film was 4 °C. As shown in Fig. 5a, the bending radius was between 17 mm and 2 mm. The relative changes in electrical conductivity and Seebeck coefficient decrease with the increase in bending radius, as illustrated in Fig. 5b and c. The properties were measured at each bending radius after 500 bending cycles. The measured changes were less than 8% compared to the pristine cases before bending, which suggest excellent flexibility and mechanical/electrical stability of the thin films. Fig. 5d and e shows the relative electrical conductivity and Seebeck coefficient changes over 1000 bending cycles at a bending radius of 4 mm, resulting in a change below 10%. The results were obtained from the film after every 100 cycles of alternate bending, up to 1000 cycles. In short, the composite thin film spray printed on Kapton demonstrated good operational durability and flexibility, opening up a potential for application in flexible TEGs.

### 3.3 Electrical properties of flexible TEGs

**Fig. 6:**
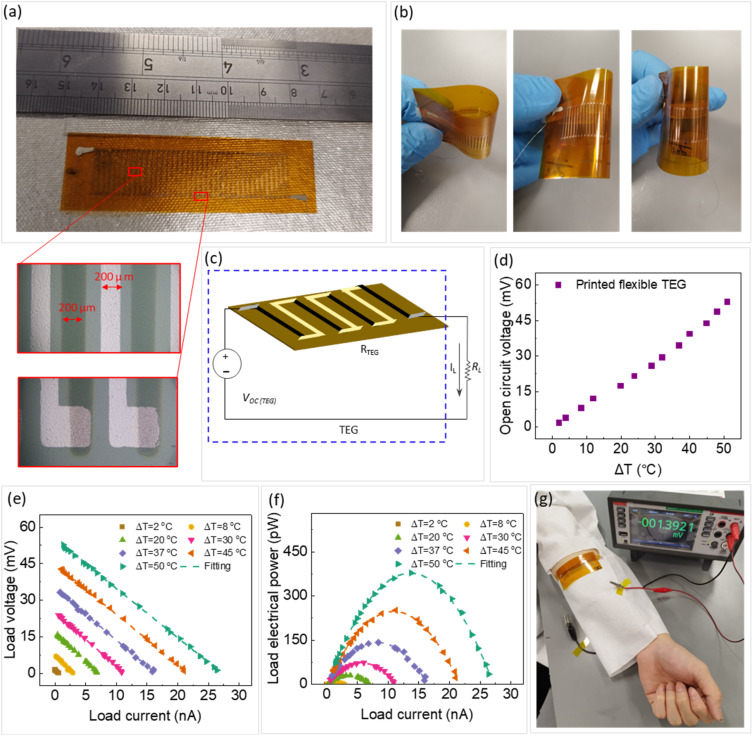
(a) Printed TEG on a Kapton substrate, (b) demonstration of TEG's flexibility, (c) equivalent circuit of the TEG, (d) the variation of open circuit voltage with temperature differences, (e and f) the variation of load voltage and output power with electrical current at different temperature differences, respectively, (g) a photograph of the printed TEG attached to a lab coat in contact with a human arm. The insets in (a) show the magnified images of fabricated TE legs and electrodes and their respective widths. The dash lines in (e and f) show the fitting of eqn (2) to the experimental data.

TEGs were fabricated on flexible Kapton substrates in four stages, including pre-treatment of the TE ink, spray printing of the TE legs, deposition of the electrodes, and wiring (more details in Section 2.3). The TE properties of the pre-treated thin film with DMSO and NaOH are presented in ESI Fig. S8.† The results show that the power factor of the composite thin film was improved by 27 folds after the pre-treatment processes with DMSO and NaOH. This can be attributed to the secondary doping and chemical dedoping of PEDOT:PSS matrix, respectively.^49^ Secondary doping modifies the PEDOT:PSS morphology by (a) segregating excess PSS and (b) inducing conformational changes in PEDOT from a benzoid to a quinoid structure.^49^ These can improve the charge transport within the structure significantly. Chemical dedoping enhances the Seebeck coefficient in PEDOT:PSS films by modifying the oxidation level of PEDOT and tuning the carrier concentration.^10^ During the dedoping process, PEDOT chains can transit from a highly oxidized bipolaron state (PEDOT^2+^) to a less oxidized polaron state (PEDOT^1+^), and even to a neutral state (PEDOT^0^). A fabricated flexible TEG on the Kapton substrate is presented in Fig. 6a. The insets in Fig. 6a show the magnified microscopic images of the TE legs and electrodes and their respective widths. The flexibility of the printed TEG is demonstrated in Fig. 6b.

Fig. S9 in ESI† illustrates images of the TE legs and metal electrodes, and their respective widths for three different TEGs fabricated. The designed width of the TE legs and electrodes was 200 μm and the designed spacing between them was 200 μm, dictated by the shadow masks used during printing (Fig. S3 in ESI†). However, due to the shadowing effect during metallization and spray printing, as well as slight misalignments of the TE leg layers, the fabricated features were wider, and the spacing between TE legs was reduced. To minimize these deviations, the shadow mask was securely fixed, spray printing parameters were optimized, and manual alignment of the electrode mask was performed. As a result, sharper TE legs, reduced shadowing effect, and spacing closer to the designed 200 μm was achieved, validating the effectiveness of these measures (Fig. S9c in ESI† and insets of Fig. 6a).

The electrical properties of the TEG were assessed using the experimental setup explained in Section 2.4. The equivalent circuit model of the TEG is shown in Fig. 6c. The open circuit voltage, load voltage, and output power of the TEG are presented in Fig. 6d–f. The open circuit voltage (*V*_OC_) of the TEG showed nearly linear behavior with respect to the temperature gradient. As shown in Fig. 6d, temperature gradients of 2 °C and 50 °C generated *V*_OC_ of ∼1.75 mV and ∼52.75 mV, respectively. The theoretical open circuit voltage of a TEG, *V*^theory^_OC_, can be calculated using the following equation:

1 *V*^theory^_OC_ = *n* × *S* × Δ*T*

where *n* is the number of TE legs, *S* is the Seebeck coefficient of a TE leg, and Δ*T* is the temperature gradient across the TEG. The Seebeck coefficient of TE leg was selected based on the average temperature across the TEG when temperature difference changed from lowest to highest values during the test. This average temperature was around 41 °C that corresponded to *S* = 30 μV K^−1^, as shown in ESI Fig. S7.† In this regard, the *V*^theory^_OC_ of the TEG with 40 legs (Fig. 6a) would be 61.2 mV at a Δ*T* of 50 °C. However, the experimental measurements of 52.7 mV for the TEG revealed a lower *V*_OC_ value, potentially due to suboptimal electrical contact (high resistance) at the junctions of the Au electrodes and TE legs, where voltage losses are likely to occur. In addition, the p-type Seebeck coefficient of the Au thin film has been reported in the range of 3–8 μV K^−1^.^58–62^ As the Au electrode is in series with the TE element and both have p-type conductivity, *V*_OC_ of the Au electrode decreases the *V*_OC_ of the TE leg.

Fig. 6e and f shows the variation of load voltage and power with electrical current at various temperature gradients for the TEG. Dashed lines were derived from the following formula for calculating the output power (*P*_out_):2
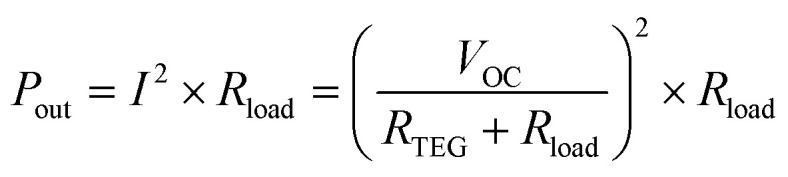
where *I* is the output current, *R*_load_ is the load resistance, and *R*_TEG_ is the internal resistance of the TEG. It is noticed in Fig. 6f that a maximum output power (*P*^max^_out_) was reached for each Δ*T*. This *P*^max^_out_ occurs when *R*_load_ is equal to *R*_TEG_, simplifying eqn (2) as following:3
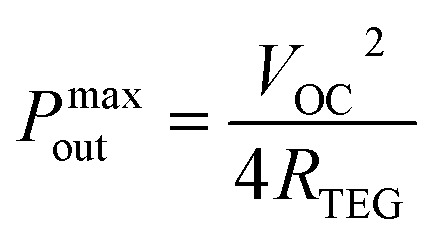


As illustrated in Fig. 6f, *P*^max^_out_ of 0.23 pW and 378 pW were obtained at Δ*T* = 2 °C and 50 °C, respectively. In these cases, *R*_load_ = *R*_TEG_ ∼ 2 MΩ. The load resistance was obtained through the fitting of eqn (2) to experimental data presented in Fig. 6f while the internal resistance of the TEG was measured using a multimeter. To assess the practicality of the fabricated flexible TEG for wearable applications, it was attached to the human arm and the *V*_OC_ was monitored, as shown Fig. 6g. Fig. S10 in ESI† provides a schematic representation of the TEG configuration on human skin, illustrating how a horizontal temperature gradient is established to enable thermoelectric energy generation. In this setup, a fabric layer insulates one side of the TEG as the “cold side”, while the opposite side contacts the skin as the “hot side”, creating a horizontal temperature gradient. The temperature gradient along the TEG was approximately 2 °C and generated a voltage of 1.39 mV.

It is noted that the output power of the printed devices in this study are lower than the state-of-the-art, as summarized in ESI Table S1.† This is primarily due to the absence of sequential post-treatment processes that can significantly change the electrical properties of PEDOT:PSS by affecting the PEDOT chains alignment and PSS concentration. For example, the authors' prior work in doping/de-doping of PEDOT:PSS/BST thin films through sequential post-treatment by H_2_SO_4_ (0.5 M) and NaOH (0.1 M) or DMSO (40 vol%) and NaOH (0.1 M) showed an increase of the power factor by up to 864- and 622-fold, respectively. In contrast, the composite thin films in this study were only pre-treated before printing by DMSO (secondary doping) and NaOH (chemical dedoping), which led to a modest 27-fold improvement in the power factor. Application of post-treatment processes to the spray-printed films and TEGs posed technical limitations. For example, when post-treatment solutions such as DMSO, H_2_SO_4_, and NaOH were spray printed on the PEDOT:PSS/BST TEGs, the TE legs got partly or fully detached from the substrate. Alternatively, manual application of the post-treatment solutions was tried, but the subsequent rinsing process led to short-circuiting of the TE legs. Despite these challenges, this study presents a pioneering effort to fabricate flexible TEGs using spray printing of organic/inorganic composites (most of the present literature has focused on fabricating flexible TEGs made of conductive polymers). The pre-treatment approach adopted here, while limited in scope, served to reduce the electrical resistance and increase the open circuit voltage of the TEGs for accurate characterization and provided a baseline for future improvements. Further investigation is planned to address these technical issues and integrate innovative post-treatment processes to achieve better performance.

## 4 Conclusions

This research focused on the development of a flexible TEG made of a PEDOT:PSS/Bi_0.5_Sb_1.5_Te_3_ composite *via* spray printing, providing an efficient solution to limitations associated with conventional fabrication techniques. Even in comparison with more commonly printed techniques such as inkjet printing, spray printing offers a more scalable fabrication process and accommodates a wider range of ink viscosities, making it suitable for depositing uniform films of various thicknesses to fabricate flexible TEGs and other electronic devices. Initially, the impact of substrate temperature on the morphological and TE properties of printed composite films was investigated. The increase in substrate temperature led to a decrease in electrical conductivity and an increase in the Seebeck coefficient due to the change in the oxidation state of PEDOT from bipolaron to polaron and neutral states. An optimum substrate temperature of 110 °C during printing enhanced the film uniformity and maximized the power factor. The flexibility, mechanical and electrical stability of the fabricated thin films were demonstrated after cyclic bending tests, showcasing their suitability for smart and wearable applications. Furthermore, a flexible TEG with 40 TE legs was fabricated using the optimized composite thin film by spray printing. The TEG exhibited excellent flexibility and generated an open circuit voltage of ∼52 mV at a temperature difference of 50 °C. Practical application was demonstrated by attaching the TEG to a human arm, monitoring the generated voltage from body heat. The TEG generated an open circuit voltage of 1.39 mV at a temperature gradient of 2 °C.

## Data availability

The data supporting this article have been included as part of the ESI.† Further information and data are available upon request from the authors.

## Author contributions

Conceptualization, A. P. and S. M.; methodology, S. M., R. G., E. C. and J. N. C.; material characterization, S. M., R. X., C. I., formal analysis, S. M.; data curation, S. M.; writing, S. M. and A. P.; supervision, A. P.; funding acquisition, A. P. All authors have given approval to the final version of the manuscript.

## Conflicts of interest

The authors declare no competing financial interest.

## Supplementary Material

RA-015-D4RA08450K-s001
