# Supplementary material for: Flexible thermoelectric generators from spray-printed PEDOT:PSS/Bi0.5Sb1.5Te3 composites
Source: RSC Adv. 2025 Feb 27;15(9):6574–84. doi: 10.1039/d4ra08450k (PMC11865908; doi:10.1039/d4ra08450k)
Supplement: RA-015-D4RA08450K-s001 [file RA-015-D4RA08450K-s001.pdf]

Supplementary Information (SI) for RSC Advances.
This journal is © The Royal Society of Chemistry 2025

Supporting Information

Flexible Thermoelectric Generators from Spray-Printed PEDOT:PSS/$Bi_{0.5}Sb_{1.5}Te_3$ Composites

Saeed Masoumi[1], Ruifeng Xiong[1], Eoin Caffrey[2,3], Riley Gatensby[2], Cansu Ilhan[2,4], Jonathan N. Coleman[2,3], and Amir Pakdel[1,*]

[1] Department of Mechanical, Manufacturing, and Biomedical Engineering, Trinity College Dublin, The University of Dublin, D02PN40, Dublin, Ireland

[2] CRANN & AMBER Research Centres, Trinity College Dublin, The University of Dublin, D02PN40, Dublin, Ireland

[3] School of Physics, Trinity College Dublin, The University of Dublin, D02PN40, Dublin, Ireland

[4] School of Chemistry, Trinity College Dublin, The University of Dublin, D02PN40, Dublin, Ireland

* Correspondence: pakdela@tcd.ie (A.P.)

Content

1. A summary of printed flexible p-type film-based TE materials and TEGs

2. Microstructural properties of BST powder after ball milling and centrifugation

3. Spray printing of PEDOT:PSS/BST composite thin films and thermoelectric generator (TEG).

4. Experimental setups for measuring electrical properties of thin films and TEGs.

5. Microstructural characterization of printed PEDOT:PSS/BST composite thin films.

6. TE properties of pre-treated PEDOT:PSS/BST composite thin films.

7. TEG fabrication and characterization

1. A summary of printed flexible p-type film-based TE materials and TEGs

Table S1. Summary of printed flexible p-type organic, inorganic, organic/inorganic composite films TE materials and devices.

	Materials	Printing method	Printing optimization	M*	Film PF ($\mu W\, m^{-1}K^{2}$)	TEG power	TEG structure/ΔT direction	Wearable use	Ref.
Inorganic	Sb_2Te_3	Dispenser	Ink formulation	-	150	nW	Coiled/Vertical	-	1
	$Bi_{0.5}Sb_{1.5}Te_3$	Dispenser	Ink formulation	-	80	µW	Circular /Horizontal	-	2
	$Bi_{0.5}Sb_{1.5}Te_3$	Inkjet	Ink formulation	-	110	nW	Planar/Horizontal	-	3
	$Bi_{0.4}5Sb_{1.55}Te_3Se_{0.03}$	Dispenser	Ink formulation	20%	2320	µW	Stack/Vertical	-	4
	$Bi_{0.5}Sb_{1.5}Te_3$	Screen	Ink formulation	10%	2400	µW	Stack/Vertical	-	5
	$Bi_{0.5}Sb_{1.5}Te_3$	Screen	Ink formulation	4%	1050	µW	Planar/Horizontal	Yes	6
Organic	PEDOT	Screen	Ink formulation	-	58.98	µW	Origami/Vertical	-	7
	PEDOT:OTf	Spray	- Ink formulation<br>- Number of layers	-	105	µW	Planar/Vertical	-	8
	PEDOT:PSS	Dispenser	Ink formulation	-	2.2	nW	Coiled/Vertical	-	9
	PEDOT:PSS	Inkjet	Ink formulation	-	17.12	nW	Coiled/Vertical	-	10
	PEDOT:PSS	Screen	Ink formulation	-	-	µW	Stack/Vertical	-	11
	PEDOT:PSS	Aerosol jet	Ink formulation	25%	2.5	nW	Planar/Horizontal	Yes	12
	PEDOT:PSS/Sb_2Te_3/Multi wall carbon nano tube (MWCNT)	Aerosol jet	- Ink formulation<br>- Hybrid atomiser	15%	41	-	-	-	13
	PEDOT:PSS/PVA	Aerosol jet	Ink formulation	1.7%	45	-	-	-	14
	PEDOT:PSS/MWCNT	Inkjet	Ink formulation	10%	7.37	nW	Planar/Horizontal	-	15
	PEDOT:PSS/ Black phosphorus	Inkjet	Ink formulation	7.53 %	13.2	-	-	-	16
Organic/inorganic composite	PEDOT:PSS/ Sb_2Te_3	Screen	Ink formulation	15%	-	µW	Planar/Horizontal	Yes	17
	PEDOT:PSS/Te	Spray	Ink formulation	-	284	nW	Planar/Horizontal	Yes	18
	PEDOT:PSS/Sb_2Te_3	Aerosol jet	- Ink formulation<br>- Number of layers	60%	28.3	µW	Coiled/Vertical	-	19
	PEDOT:PSS/Te	Screen	Ink formulation	-	4.9	nW	Radial/Vertical	-	20

	PEDOT:PSS/Cu_2S	Screen	-	25%	20.6	nW	Radial/Vertical	-	[21]
	PEDOT:PSS/ $Bi_{0.5}Sb_{1.5}Te_3$	Spray	- Ink formulation<br>- Substrate temperature	10%	4	pW	Planar/Horizontal	Yes	This work
	M* : Percentage of electrical properties degradation after mechanical deformation (Bending radius and cycle)								

It is noted that the output power of the printed devices in this study are lower than the state-of-the-art, as summarized in Supporting Information **Table S1**. This is primarily due to the absence of sequential post-treatment processes that can significantly change the electrical properties of PEDOT:PSS by affecting the PEDOT chains alignment and PSS concentration. For example, the authors' prior work in doping/de-doping of PEDOT:PSS/BST thin films through sequential post-treatment by H_2SO_4 (0.5 M) and NaOH (0.1 M) or DMSO (40 vol%) and NaOH (0.1 M) showed an increase of the power factor by up to 864- and 622-fold, respectively. In contrast, the composite thin films in this study were only pre-treated before printing by DMSO (secondary doping) and NaOH (chemical dedoping), which led to a modest 27-fold improvement in the power factor. Application of post-treatment processes to the spray-printed films and TEGs posed technical limitations. For example, when post-treatment solutions such as DMSO, H_2SO_4, and NaOH were spray printed on the PEDOT:PSS/BST TEGs, the TE legs got partly or fully detached from the substrate. Alternatively, manual application of the post-treatment solutions was tried, but the subsequent rinsing process led to short-circuiting of the TE legs.

2. Microstructural properties of BST powder after ball milling and centrifugation

Figure S1 displays SEM micrographs and particle distribution obtained after ball milling and centrifugation processes. Ball milling resulted in average particle sizes of 0.99 μm. Centrifugation yielded the average particle sizes of 1.389 μm at speeds of 500 rpm. This increase in particle size is attributed to the separation process during centrifugation, where smaller particles remain in the liquid above, while larger particles settle due to their higher mass and density. The centrifugal force at 500 rpm selectively concentrates the larger BST particles, leading to a higher average particle size.

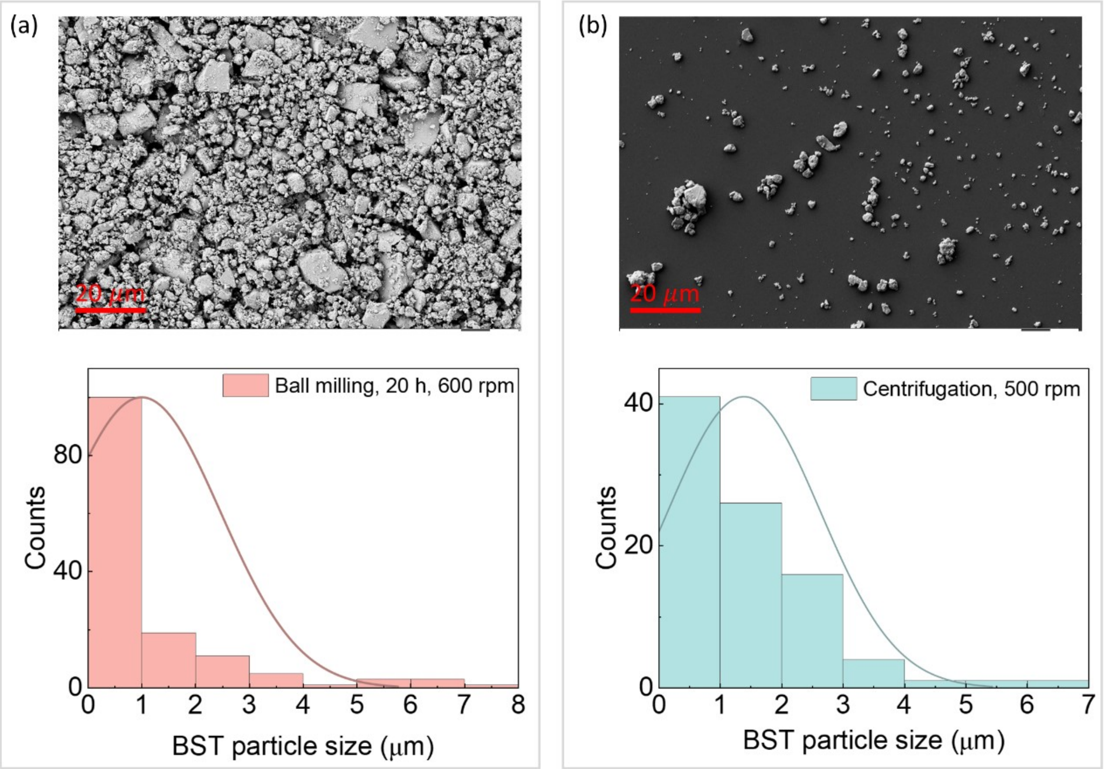

Figure S1. SEM micrographs and particle size distribution of the BST powder after (a) ball milling and (b) centrifugation.

3. Spray printing of PEDOT:PSS/BST composite thin films and thermoelectric generators (TEGs)

Figure S2a displays photos of the PEDOT:PSS/BST-based TE ink used in the spray printing process. In **Figure S2b**, the spray printing machine is depicted, featuring the nozzle, ink container, and hot plate. The insets in **Figure S2b** provide a closer look at the mounted glass substrate on a hot plate for the printing process and the nozzle of the printing machine. Thin films printed on the glass substrate at temperatures of 40 °C, 80 °C, 110 °C, and 150 °C are presented in **Figure S2c-f**, respectively. **Figure S2g, h** showcases the composite thin film printed on a Kapton substrate at a temperature of 110 °C, and demonstrating its flexibility.

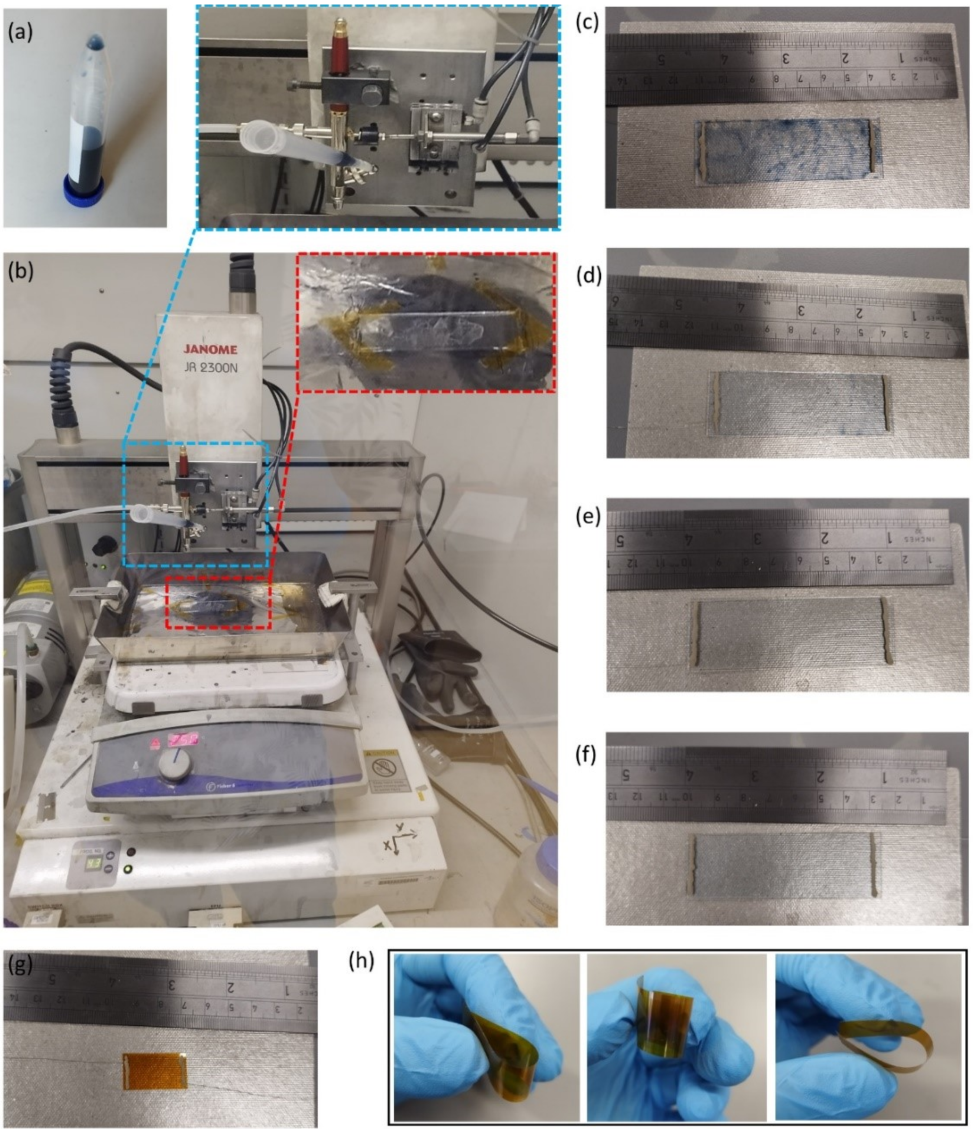

Figure S2. The photos of (a) ink, (b) spray printing machine, printed films on glass substrate at the temperature of (c) 40 ℃, (d) 80 ℃, (e) 110 ℃, and (f) 150 ℃, (g) Kapton substrate at the temperature of 110 ℃, and (h) flexibility of printed films on Kapton substrate. The insets in (b) show the mounted glass substrate on a hot plate for printing process and nozzle of printing machine.

Additionally, **Figure S3a, b** illustrates the shadow masks used for fabricating the TE legs and metal electrodes to construct a TEG, respectively.

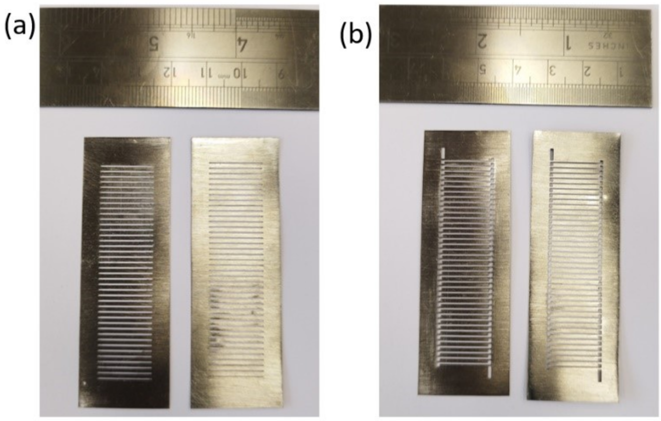

Figure S3. Shadow masks for (a) TE legs and (b) metal electrodes.

4. Experimental setups for measuring electrical properties of thin films and TEGs

Figure S4 presents a photograph of a home-made apparatus designed for measuring the electrical resistance and Seebeck coefficient of thin film samples. Two independent microheaters were employed to create temperature differences across the samples. For temperature measurement on the hot and cold sides of the samples, two K-type thermocouples were utilized. Analysis of the current-voltage (I-V) patterns facilitated the determination of electrical resistance and Seebeck voltage in the thin film samples by measuring the slope and intercept of the I-V characteristic, respectively. To facilitate this process, a graphical user interface and an interface circuit were developed [22-25].

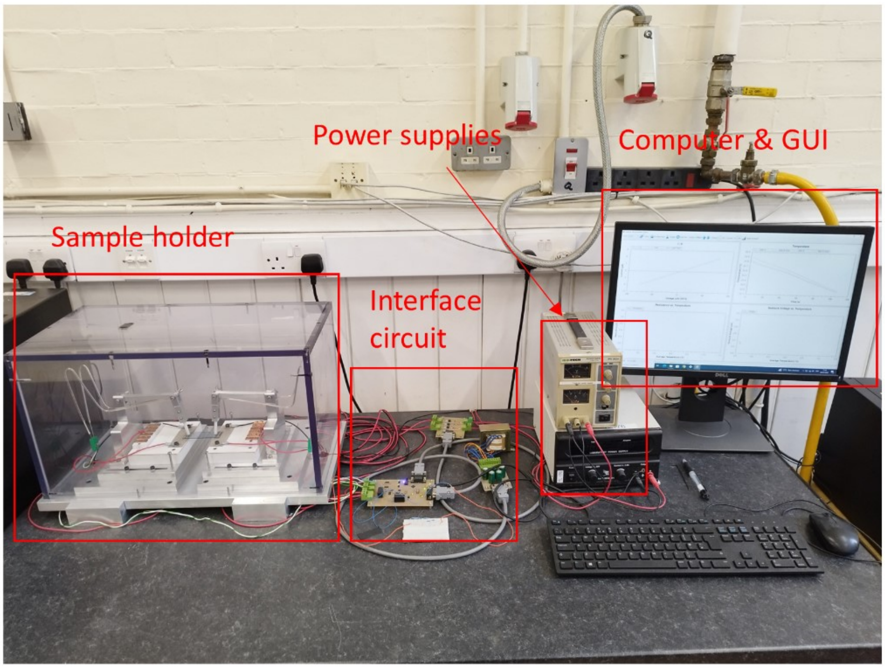

Figure S4. An experimental setup for electrical characterization of TE thin film samples.

Figure S5 displays a custom experimental setup designed to assess the electrical performance of a TEG. This setup incorporates two commercially available Peltier modules, each sized at 4 cm × 4 cm, to heat one side of the TEG. Temperature differences along the TEG were measured using two K-type thermocouples. The open circuit voltage of the TEG was measured at various temperature differences using a digital multimeter (Keithley, DMM6500). To achieve impedance matching at each measurement temperature, the TEG was connected to a load shunt variable resistor (General Radio Co/Decade resistor, variable resistance box). The current and output power at each measurement temperature were calculated using the measured load voltage and shunt resistance.

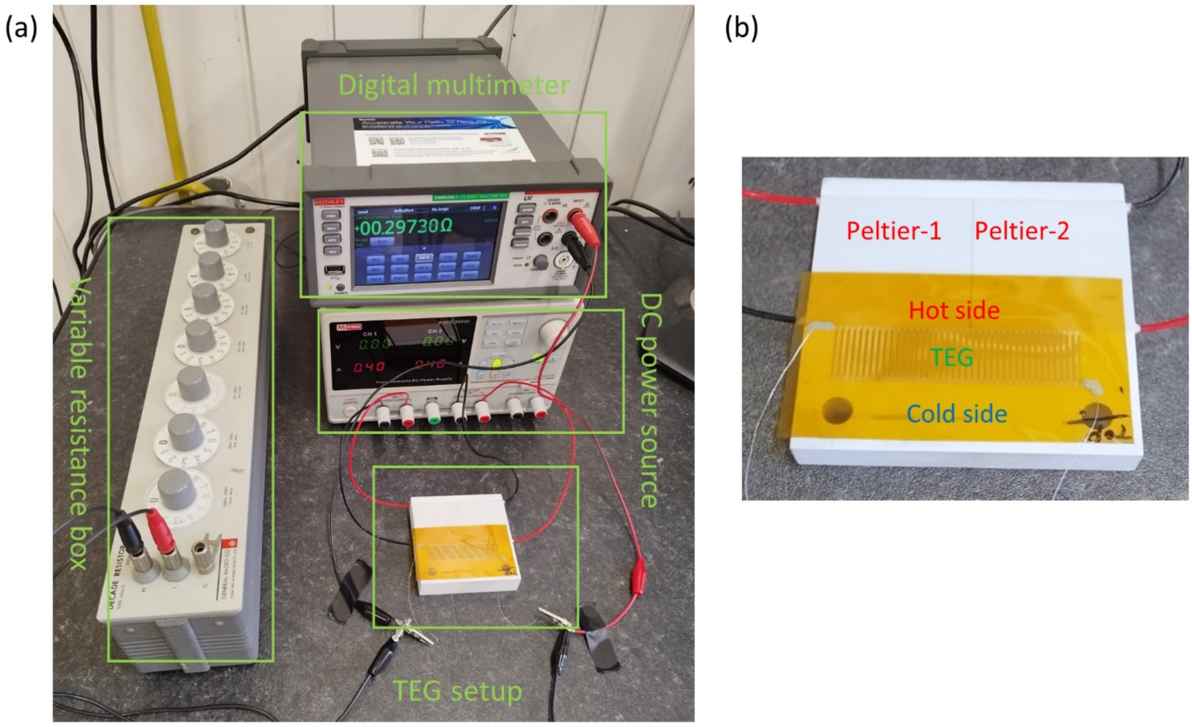

Figure S5. (a) An experimental setup for electrical characterization of TEGs, and (b) the test bed made of Peltier devices for providing temperature differences along the TEGs on flexible Kapton substrate.

5. Microstructural characterization of printed PEDOT:PSS/BST thin films

Figure S6 illustrates a typical step-height profile for PEDOT:PSS/BST composite thin films printed at various temperatures, namely 40 °C, 80 °C, 110 °C, and 150 °C. There was not a significant change in the average thickness of the composite films printed above 80 °C, maintaining a range of 108-145 nm. However, the thin film printed at 40 °C had a considerably higher average thickness. **Figure S7** shows typical SEM micrographs of PEDOT:PSS thin films containing 0 and 40 wt% BST particles. It is evident that particle distribution is homogenous in the composite sample.

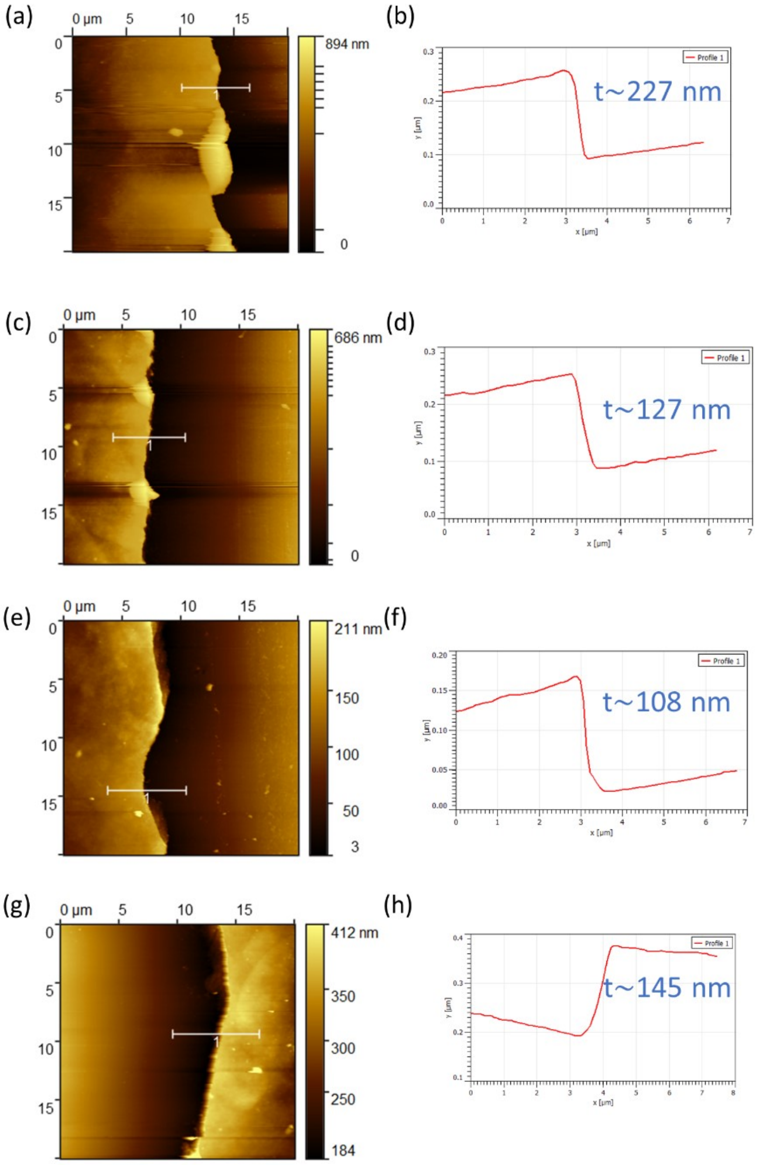

Figure S6. Typical step-height profile (film thickness (t)) for PEDOT:PSS/BST composite thin films printed at different temperatures, (a, b) 40 ℃, (c, d) 80 ℃, (e, f) 110 ℃, (g, h) 150 ℃.

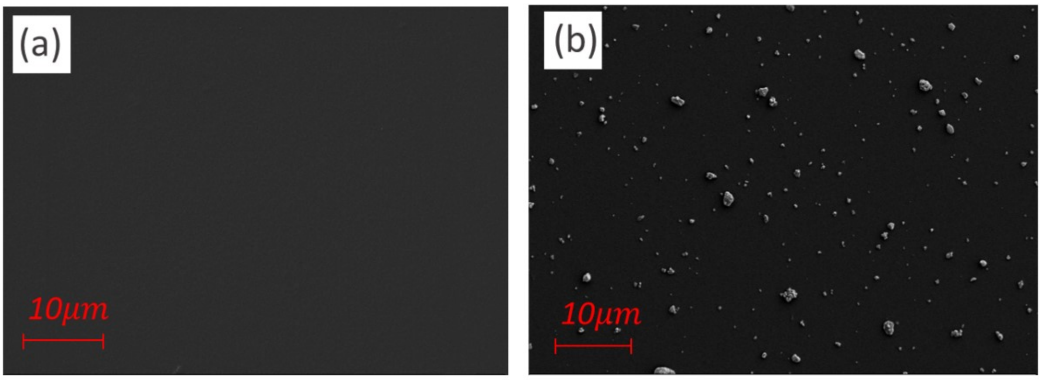

Figure S7. SEM micrographs of PEDOT:PSS thin films with (a) 0, and (b) 40 wt% BST, respectively.

6. Thermoelectric properties of pre-treated PEDOT:PSS/BST thin films

The TE properties of PEDOT:PSS/BST thin films were enhanced through pre-treatment of the ink with 10% vol. of DMSO and NaOH (0.1 M of NaOH) before the spray printing process, as detailed in Section 2.3 of the manuscript. Electrical measurements were conducted following the procedure outlined in Section 2.4. **Figure S8** illustrates the temperature dependence of electrical conductivity, Seebeck coefficient, and power factor of the pre-treated thin film. The results indicate a decrease in electrical conductivity with temperature, concurrent with an increase in the Seebeck coefficient. This observation suggests metallic or semi-metallic transport behaviour in the pre-treated sample. Furthermore, the power factor of the thin film improved by 27 times after the treatment process.

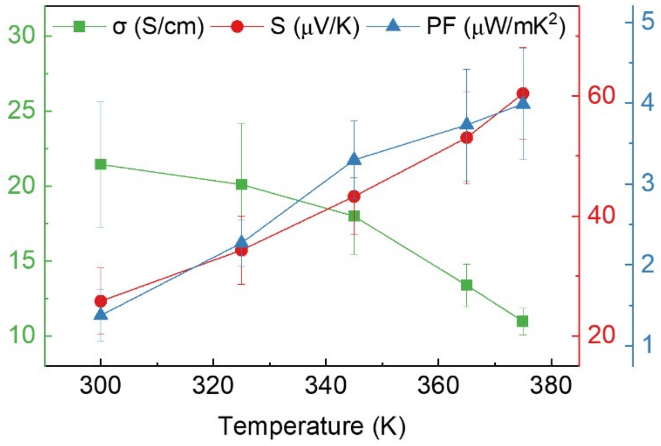

Figure S8. Temperature dependency of electrical conductivity, Seebeck coefficient, and power factor of pre-treated PEDOT:PSS/BST thin film fabricated by spray printing. The error bars were calculated by two times repetition of measurements on each sample.

The reported electrical conductivity of DMSO-treated PEDOT:PSS samples in literature varies widely, between 12 and 619 S/cm [26-33]. This discrepancy can be attributed to several factors, including the DMSO concentration, co-treatment with a dedoping agent (e.g. NaOH), thermal treatment duration, fabrication method, PEDOT:PSS product grade, and the film's thickness. As an example, different PEDOT:PSS grades, such as Clevios PH1000, Clevios PH510, and Sigma-Aldrich, exhibit varying properties due to differences in PEDOT:PSS ratios, solid content, and baseline conductivities [34].

While a direct comparison between sequentially post-treated samples by DMSO and the pre-treated samples in the present research cannot be made, **Figure S8** shows that the pre-treated thin films exhibited a maximum electrical conductivity of 22 S/cm and a power factor of 4 μW/mK^2, demonstrating a significant improvement compared to the untreated films (i.e., σ = 4 S/cm, S = 0.2 μW/mK^2). These lower values in pre-treated samples as compared to post-treated ones may be attributed to the more effective removal of insulating PSS, stronger polymer chain alignment, and greater phase separation in the post-treatment process. In case of sequential post-treatment, DMSO and NaOH

solutions are applied to already-fabricated PEDOT:PSS films, allowing for better PSS removal from the surface and grain boundaries. However, in the case of pre-treatment, the doping/dedoping agents are added to the PEDOT:PSS solution before deposition, leading to partial PSS dissolution and removal; thus more PSS remains in the film during drying. Post-treatment reorganizes PEDOT chains into a more linear, extended conformation after solidification, enhancing π-π stacking for better charge mobility, whereas pre-treatment is limited by rapid solvent evaporation that prevents full alignment of the chains. Furthermore, sequential post-treatment effectively enhances phase separation in multiple rounds by pushing PSS out of PEDOT-rich regions, creating a more interconnected conductive network, while a single-round pre-treatment process yields less controlled phase separation, leaving insulating PSS randomly distributed within the film.

7. TEG fabrication and characterization

Figure S9 in Supporting Information illustrates images of the TE legs and metal electrodes, and their respective widths for three different TEGs fabricated. The designed width of the TE legs and electrodes was 200 µm and the designed spacing between them was 200 µm, controlled by the shadow masks used during printing (Figure S3 in Supporting Information). However, due to the shadowing effect during metallization and spray printing, as well as slight misalignments of the TE leg layers, the fabricated features were wider, and the spacing between TE legs was reduced. To minimize these deviations, the shadow mask was securely fixed, spray printing parameters were optimized, and manual alignment of the electrode mask was performed. As a result, sharper TE legs, reduced shadowing effect, and spacing closer to the designed 200 µm was achieved, validating the effectiveness of these measures (Figure S9c in Supporting Information and insets of Figure 6a).

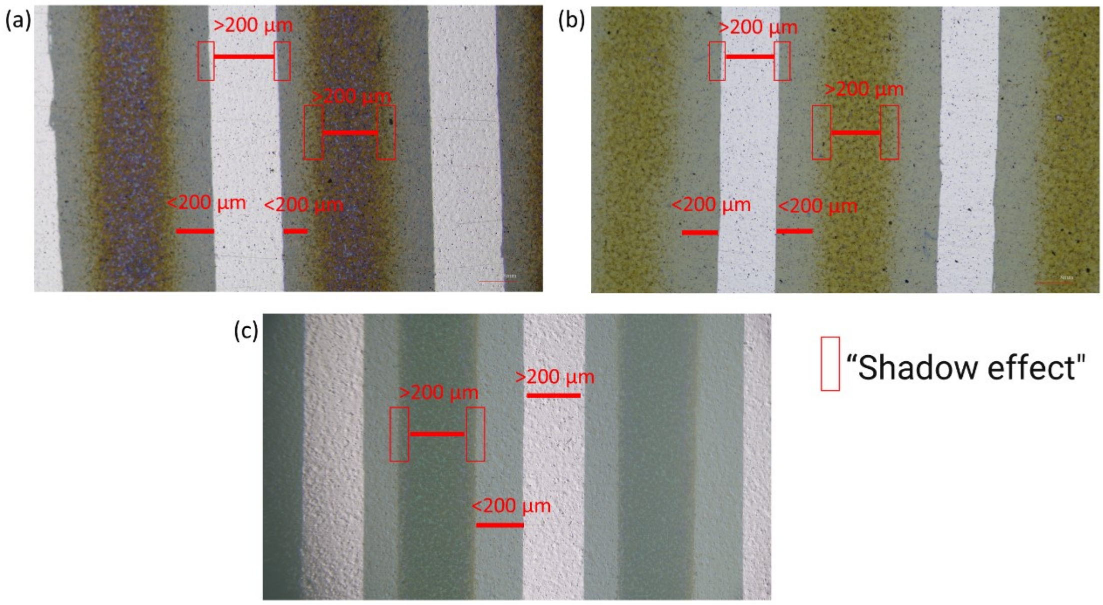

Figure S9. Images of printed TE legs, deposited electrodes, and their respective widths for three different TEGs on Kapton substrates: (a) TEG-1, (b) TEG-2, and (c) TEG-3.

Figure S10 provides a schematic representation of the TEG configuration on human skin, illustrating how a horizontal temperature gradient is established to enable thermoelectric energy generation. In this setup, one side of the TEG is placed on a layer of fabric, which acts as a thermal insulator. This fabric layer minimizes heat transfer between the TEG and the skin, maintaining a lower temperature on this side of the TEG, effectively creating a "cold side". The opposite side of the TEG is in direct contact with the skin, which serves as the "hot side" due to the heat emanating from the body.

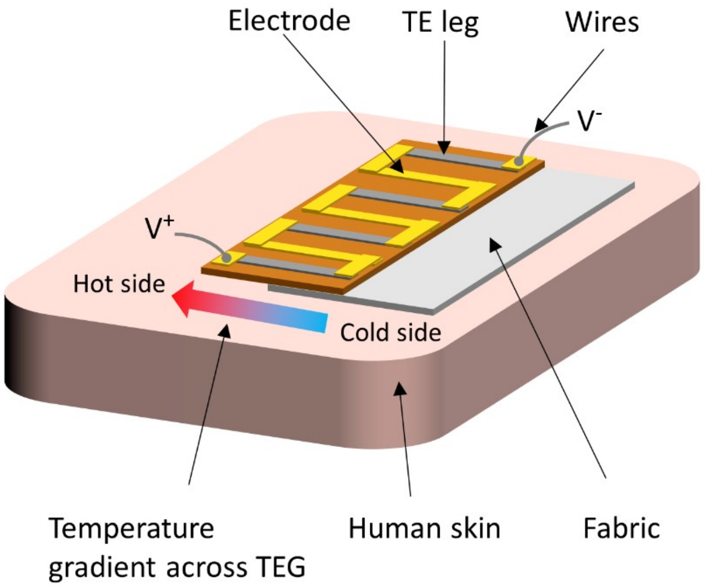

Figure S10. A schematic presentation of the printed TEG on human skin, illustrating the horizontal temperature gradient established across it.

References

1. Chen, A.; Madan, D.; Wright, P.; Evans, J., Dispenser-printed planar thick-film thermoelectric energy generators. *Journal of Micromechanics and Microengineering* **2011,** *21* (10), 104006.
2. Madan, D.; Wang, Z.; Chen, A.; Winslow, R.; Wright, P. K.; Evans, J. W., Dispenser printed circular thermoelectric devices using Bi and Bi0. 5Sb1. 5Te3. *Applied Physics Letters* **2014,** *104* (1).
3. Chen, B.; Kruse, M.; Xu, B.; Tutika, R.; Zheng, W.; Bartlett, M. D.; Wu, Y.; Claussen, J. C., Flexible thermoelectric generators with inkjet-printed bismuth telluride nanowires and liquid metal contacts. *Nanoscale* **2019,** *11* (12), 5222-5230.
4. Chien, S.-Y.; Hou, L.-C.; Li, C.-C.; Liao, C.-N., Flexible thermoelectric generators prepared by dispenser printing technology. *Materials Chemistry and Physics* **2022,** *287*, 126269.
5. Mallick, M. M.; Franke, L.; Rösch, A. G.; Geßwein, H.; Long, Z.; Eggeler, Y. M.; Lemmer, U., High Figure-of-Merit Telluride-Based Flexible Thermoelectric Films through Interfacial Modification via Millisecond Photonic-Curing for Fully Printed Thermoelectric Generators. *Advanced Science* **2022,** *9* (31), 2202411.
6. Shi, J.; Wu, X.; Geng, X.; Hu, L.; Liu, F.; Ao, W.; Zhang, C., Anisotropy engineering in solution-derived nanostructured Bi2Te3 thin films for high-performance flexible thermoelectric devices. *Chemical Engineering Journal* **2023,** *458*, 141450.
7. Rösch, A. G.; Gall, A.; Aslan, S.; Hecht, M.; Franke, L.; Mallick, M. M.; Penth, L.; Bahro, D.; Friderich, D.; Lemmer, U., Fully printed origami thermoelectric generators for energy-harvesting. *npj Flexible Electronics* **2021,** *5* (1), 1.
8. Yvenou, E.; Sandroni, M.; Carella, A.; Gueye, M. N.; Faure-Vincent, J.; Pouget, S.; Demadrille, R.; Simonato, J.-P., Spray-coated PEDOT: OTf films: thermoelectric properties and integration into a printed thermoelectric generator. *Materials Chemistry Frontiers* **2020,** *4* (7), 2054-2063.
9. Stepien, L.; Roch, A.; Schlaier, S.; Dani, I.; Kiriy, A.; Simon, F.; Lukowicz, M. v.; Leyens, C., Investigation of the thermoelectric power factor of KOH-treated PEDOT: PSS dispersions for printing applications. *Energy Harvesting and Systems* **2016,** *3* (1), 101-111.
10. Ferhat, S.; Domain, C.; Vidal, J.; Noël, D.; Ratier, B.; Lucas, B., Organic thermoelectric devices based on a stable n-type nanocomposite printed on paper. *Sustainable Energy & Fuels* **2018,** *2* (1), 199-208.
11. Mukaida, M.; Wei, Q.; Ishida, T., Polymer thermoelectric devices prepared by thermal lamination. *Synthetic Metals* **2017,** *225*, 64-69.
12. Kee, S.; Haque, M. A.; Corzo, D.; Alshareef, H. N.; Baran, D., Self-healing and stretchable 3D-printed organic thermoelectrics. *Advanced Functional Materials* **2019,** *29* (51), 1905426.
13. Ou, C.; Sangle, A. L.; Chalklen, T.; Jing, Q.; Narayan, V.; Kar-Narayan, S., Enhanced thermoelectric properties of flexible aerosol-jet printed carbon nanotube-based nanocomposites. *Apl Materials* **2018,** *6* (9).
14. Du, Y.; Chen, J.; Qin, J.; Meng, Q.; Shen, S. Z., Flexible PVA/PEDOT: PSS thermoelectric nanocomposite films prepared via an additive manufacturing process. *Composites Communications* **2022,** *35*, 101312.
15. Li, H.; Zhou, S.; Han, S.; Luo, R.; Hu, J.; Du, B.; Yang, K.; Bao, Y.; Jia, J.; Zhang, X., Thermoelectric Properties of One-Pot Hydrothermally Synthesized Solution-Processable PEDOT:PSS/MWCNT Composite Materials. *Polymers (Basel)* **2023,** *15* (18), 3781.
16. Xiao, C.; Liu, X.; Meng, Q.; Wang, L.; Du, Y.; Li, Z., Fabrication of flexible self-supporting black phosphorus nanosheet/PEDOT: PSS thermoelectric composite films by solution 3D printing technology. *Composites Communications* **2024,** *46*, 101815.
17. We, J. H.; Kim, S. J.; Cho, B. J., Hybrid composite of screen-printed inorganic thermoelectric film and organic conducting polymer for flexible thermoelectric power generator. *Energy* **2014,** *73*, 506-512.

18. Jin Bae, E.; Hun Kang, Y.; Jang, K.-S.; Yun Cho, S., Enhancement of thermoelectric properties of PEDOT: PSS and tellurium-PEDOT: PSS hybrid composites by simple chemical treatment. *Scientific reports* **2016,** *6* (1), 18805.
19. Ou, C.; Sangle, A. L.; Datta, A.; Jing, Q.; Busolo, T.; Chalklen, T.; Narayan, V.; Kar-Narayan, S., Fully Printed Organic-Inorganic Nanocomposites for Flexible Thermoelectric Applications. *ACS Appl Mater Interfaces* **2018,** *10* (23), 19580-19587.
20. Menon, A. K.; Meek, O.; Eng, A. J.; Yee, S. K., Radial thermoelectric generator fabricated from n-and p-type conducting polymers. *Journal of Applied Polymer Science* **2017,** *134* (3).
21. Zhao, J.; Zhao, X.; Guo, R.; Zhao, Y.; Yang, C.; Zhang, L.; Liu, D.; Ren, Y., Preparation and Characterization of Screen-Printed Cu(2)S/PEDOT:PSS Hybrid Films for Flexible Thermoelectric Power Generator. *Nanomaterials (Basel)* **2022,** *12* (14), 2430.
22. Masoumi, S.; Noori, A.; Pakdel, A., Exploring electrical transport in thin film and bulk thermoelectric materials with an automated Seebeck coefficient and resistivity measurement platform. *Measurement* **2024**, 115162.
23. Xiong, R.; Masoumi, S.; Pakdel, A., An Automatic Apparatus for Simultaneous Measurement of Seebeck Coefficient and Electrical Resistivity. *Energies* **2023,** *16* (17), 6319.
24. Masoumi, S.; Noori, A.; Shokrani, M.; Hossein-Babaei, F., Apparatus for seebeck coefficient measurements on high-resistance bulk and thin-film samples. *IEEE Transactions on Instrumentation and Measurement* **2019,** *69* (6), 3070-3077.
25. Hossein-Babaei, F.; Masoumi, S.; Noori, A., Seebeck voltage measurement in undoped metal oxide semiconductors. *Measurement Science and Technology* **2017,** *28* (11), 115002.
26. Alam, J.; Xu, X.; Adu, P. C. O.; Meng, Q.; Zuber, K.; Afshar, S.; Kuan, H.-C.; Ma, J., Enhancing thermoelectric performance of PEDOT: PSS: A review of treatment and nanocomposite strategies. *Advanced Nanocomposites* **2024,** *1* (1), 16-38.
27. Lim, K.; Jung, S.; Lee, S.; Heo, J.; Park, J.; Kang, J.-W.; Kang, Y.-C.; Kim, D.-G., The enhancement of electrical and optical properties of PEDOT: PSS using one-step dynamic etching for flexible application. *Organic Electronics* **2014,** *15* (8), 1849-1855.
28. Kim, J.; Jung, J.; Lee, D.; Joo, J., Enhancement of electrical conductivity of poly (3, 4-ethylenedioxythiophene)/poly (4-styrenesulfonate) by a change of solvents. *Synthetic Metals* **2002,** *126* (2-3), 311-316.
29. Jönsson, S.; Birgerson, J.; Crispin, X.; Greczynski, G.; Osikowicz, W.; Van Der Gon, A. D.; Salaneck, W. R.; Fahlman, M., The effects of solvents on the morphology and sheet resistance in poly (3, 4-ethylenedioxythiophene)–polystyrenesulfonic acid (PEDOT–PSS) films. *Synthetic metals* **2003,** *139* (1), 1-10.
30. Feng-Xing, J.; Jing-Kun, X.; Bao-Yang, L.; Yu, X.; Rong-Jin, H.; Lai-Feng, L., Thermoelectric performance of poly (3, 4-ethylenedioxythiophene): poly (styrenesulfonate). *Chinese Physics Letters* **2008,** *25* (6), 2202.
31. Ouyang, J.; Xu, Q.; Chu, C.-W.; Yang, Y.; Li, G.; Shinar, J., On the mechanism of conductivity enhancement in poly (3, 4-ethylenedioxythiophene): poly (styrene sulfonate) film through solvent treatment. *Polymer* **2004,** *45* (25), 8443-8450.
32. Liu, C.; Lu, B.; Yan, J.; Xu, J.; Yue, R.; Zhu, Z.; Zhou, S.; Hu, X.; Zhang, Z.; Chen, P., Highly conducting free-standing poly (3, 4-ethylenedioxythiophene)/poly (styrenesulfonate) films with improved thermoelectric performances. *Synthetic Metals* **2010,** *160* (23-24), 2481-2485.
33. Yi, C.; Wilhite, A.; Zhang, L.; Hu, R.; Chuang, S. S.; Zheng, J.; Gong, X., Enhanced thermoelectric properties of poly (3, 4-ethylenedioxythiophene): poly (styrenesulfonate) by binary secondary dopants. *ACS applied materials & interfaces* **2015,** *7* (17), 8984-8989.
34. Shi, H.; Liu, C.; Jiang, Q.; Xu, J., Effective approaches to improve the electrical conductivity of PEDOT: PSS: a review. *Advanced Electronic Materials* **2015,** *1* (4), 1500017.
35. Luo, J.; Billep, D.; Waechtler, T.; Otto, T.; Toader, M.; Gordan, O.; Sheremet, E.; Martin, J.; Hietschold, M.; Zahn, D. R., Enhancement of the thermoelectric properties of PEDOT: PSS thin films by post-treatment. *Journal of Materials Chemistry A* **2013,** *1* (26), 7576-7583.

36. Unsworth, N. K.; Hancox, I.; Dearden, C. A.; Sullivan, P.; Walker, M.; Lilley, R.; Sharp, J.; Jones, T. S., Comparison of dimethyl sulfoxide treated highly conductive poly (3, 4-ethylenedioxythiophene): poly (styrenesulfonate) electrodes for use in indium tin oxide-free organic electronic photovoltaic devices. *Organic Electronics* **2014,** *15* (10), 2624-2631.
37. Tu, S.; Tian, T.; Oechsle, A. L.; Yin, S.; Jiang, X.; Cao, W.; Li, N.; Scheel, M. A.; Reb, L. K.; Hou, S., Improvement of the thermoelectric properties of PEDOT: PSS films via DMSO addition and DMSO/salt post-treatment resolved from a fundamental view. *Chemical Engineering Journal* **2022,** *429*, 132295.
